# Characterization of global 5-hydroxymethylcytosine in pediatric posterior fossa ependymoma

**DOI:** 10.1186/s13148-020-0809-8

**Published:** 2020-01-28

**Authors:** Tao Wu, Zhi-wei Zhang, Shiwei Li, Bo Wang, Zhijun Yang, Peng Li, Jing Zhang, Wei-min Tong, Chunde Li, Fu Zhao, Yamei Niu, Pinan Liu

**Affiliations:** 1 Department of Neurosurgery, Beijing Tiantan Hospital, Capital Medical University, 119# South 4th Ring Road, Fengtai District, Beijing, 100070 China; 2 Department of Neural reconstruction, Beijing Neurosurgical Institute, Capital Medical University, No. 119, South 4th Ring Road, Fengtai District, Beijing, 100070 China; 3 Department of Pathology, Institute of Basic Medical Sciences Chinese Academy of Medical Science, School of Basic Medicine Peking Union Medical College; Molecular Pathology Research Center, Chinese Academy of Medical Sciences, Beijing, 100005 China

**Keywords:** Pediatric ependymoma, Molecular subgroup, 5-Hydroxymethylcytosine, Epigenetics, Ki-67 index, Prognosis, 1q gain

## Abstract

**Background:**

5-Hydroxymethylcytosine (5hmC) is a novel epigenetic mark and may be involved in the mechanisms of tumorigenesis and malignant transformation. However, the role of 5hmC in ependymoma, the third most common brain tumor in children, remains unclear. The aim of this study sought to identify the characterization of 5hmC levels in pediatric posterior fossa ependymoma and to evaluate whether 5hmC levels could be a potential factor to predict clinical outcomes.

**Results:**

Our results showed that 5hmC levels were globally decreased in posterior fossa ependymoma compared with normal cerebellum tissues (*P* < 0.001). Group A posterior fossa ependymomas had higher 5hmC levels than group B tumors (*P* = 0.007). Moreover, 5hmC levels positively correlated with Ki-67 index in posterior fossa ependymoma (*r* = 0.428, *P* = 0.003). Multivariate Cox hazards model revealed that patients with high 5hmC levels (> 0.102%) had worse PFS and OS than patients with lower 5hmC levels (< 0.102%) (PFS: HR = 3.014; 95% CI, 1.040–8.738; *P* = 0.042; OS: HR = 2.788; 95% CI, 0.974–7.982; *P* = 0.047).

**Conclusions:**

Our findings suggest that loss of 5hmC is an epigenetic hallmark for pediatric posterior fossa ependymoma. 5hmC levels may represent a potential biomarker to predict prognosis in children with posterior fossa ependymoma.

## Background

Ependymoma (EPN) is a relatively rare neuroepithelial tumor that arises throughout the whole neuraxis [1]. Intracranial EPN predominantly occurs in children and adolescents, with two third of those tumors located in posterior fossa [1, 2]. Recently, posterior fossa ependymoma (EPN_PF) has been classified into two molecular subgroups based on DNA CpG island (CpGi) methylation profiles status [3–6]. Group A ependymoma (EPN_PFA) is characterized by CpGi hypermethylation with 1q gain and occurs predominantly in infancy and young children. These subgroup tumors also exhibit global low H3K27me3 [7–9], global DNA hypomethylation [7], and high expression of *EZHIP* [10]. Conversely, group B ependymoma (EPN_PFB) presents with CpGi hypomethylation and primarily occurs in adolescences and young adults. Moreover, the molecular classification of EPN has provided a superior prognostic prediction and risk stratification [11]. EPN_PFA tumors are often difficult to completely resect and bear a dismal prognosis, while EPN_PFB tumors are less invasive and carry a favorable prognosis [4, 5]. It suggests that epigenetic mechanisms play an essential role in EPN_PF pathogenesis and tumor maintenance.

Abnormal DNA methylation at the 5 position of cytosine (5mC) is an epigenetic mark of cancers. Recent studies presented evidence for an active DNA demethylation pathway initiated by the ten-eleven translocation (TET) protein family, resulting in the conversion of 5mC into 5-hydroxymethylcytosine (5hmC) [12, 13]. As a new epigenetic biomarker, 5hmC is reshaping the view of the tumor epigenome. Several reports have shown that decreased 5hmC level is an indicator of poor survival in the central nervous system (CNS) tumors patients [14–17]. However, only one report studied the changes of 5hmC as well as its downstream products in two EPN cell lines, which represent a subgroup of supratentorial EPN with *RELA* fusion [18].

In the present study, we performed the ultra-high-performance liquid chromatography-mass spectrometry (UHPLC-MS/MS) analysis and immunochemistry (IHC) staining analysis to measure global 5hmC and 5mC levels to relate this information to clinical characteristics and survival outcomes in pediatric EPN_PF.

## Results

### Clinical characteristics

Forty-five cases of pathologically WHO grades II/III confirmed EPN_PF (age < 18) treated in Beijing Tiantan Hospital between Jan 2010 to Dec 2017 were identified. The clinical data of the institutional cohort were summarized in Table 1. Median age at diagnosis of these children was 4 years (range 1–17). The male to female ratio was 2.8:1 (33/12). The maximum diameter of tumor ranged from 2.3 to 19.5 cm with a median size of 4.7 cm.

**Table 1:** Clinical characteristics of pediatric posterior fossa ependymoma

Characteristics	Value (%)
Demographics	
No. of patients	45
Age, years, median (range)	4 (1–17)
Gender, (female/male)	12/33
Tumor size, cm, median (range)	4.7 (2.3–19.5)
Extent of surgery, GTR, *n* (%)	21 (46.7)
Radiotherapy, yes, *n* (%)	29 (64.4)
Chemotherapy, yes, *n* (%)	12 (26.7)
Histology, *n* (%)	
WHO II	10 (22.2)
WHO III	35 (77.8)
Ki-67 index, *n* (%)	
< 20%	20 (44.4)
≥ 20%	25 (55.6)
Molecular subgroups, *n* (%)	
EPN_PFA	35 (77.8)
EPN_PFB	10 (22.2)
Chromosome 1q	
Gain	16 (35.6)
No gain	29 (64.4)
5hmC/(C + mC) × 100%, median (range)	0.127 (0.028–0.341)
5mC/(C + mC) × 100%, mean ± SD	3.664 ± 0.426
Recurrence, *n* (%)	25 (55.6)
Death during follow-up, *n* (%)	23 (51.1)
Follow-up period, months, median (range)	38 (6–60)

*GTR* gross total resection, *5hmC* 5- hydroxymethylcytosine, 5*mC* 5-methylcytosine, *C* cytosine, *EPN_PFA* Group A posterior fossa ependymoma, *EPN_PFB* Group B posterior fossa ependymoma

Gross total resection (GTR) was achieved in 21 (46.7%) of patients while 24 (53.3%) had a subtotal resection (STR). Histopathological diagnosis presented ten (22.2%) patients with EPN of WHO grade II and 35 (77.8%) patients with EPN of WHO grade III. We performed immunostaining of H3K27me3 to distinguish EPN_PFA from EPN_PFB (Additional file 1: Figure S1A). We found that 35 of 45 (77.8%) were negative for H3K27me3 staining and designated as EPN_PFA, while 10 of 45 (22.2%) were positive as PFB (Additional file 1: Figure S1B). EPN_PFA patients were much younger than EPN_PFB patients (*P* < 0.001, Additional file 1: Figure S1C). Interphase fluorescence in situ hybridization (FISH) analysis revealed that 16 tumors (35.6%) had chromosome 1q25 gain, while 29 tumors (64.4%) had a balanced chromosome 1 (Additional file 1: Figure S2A, B). A total of 29 (64.4%) patients were treated with postoperative focal radiotherapy, and 12 (26.7%) patients received chemotherapy.

### 5hmC levels were decreased in EPN_PF

**Fig. 1:**
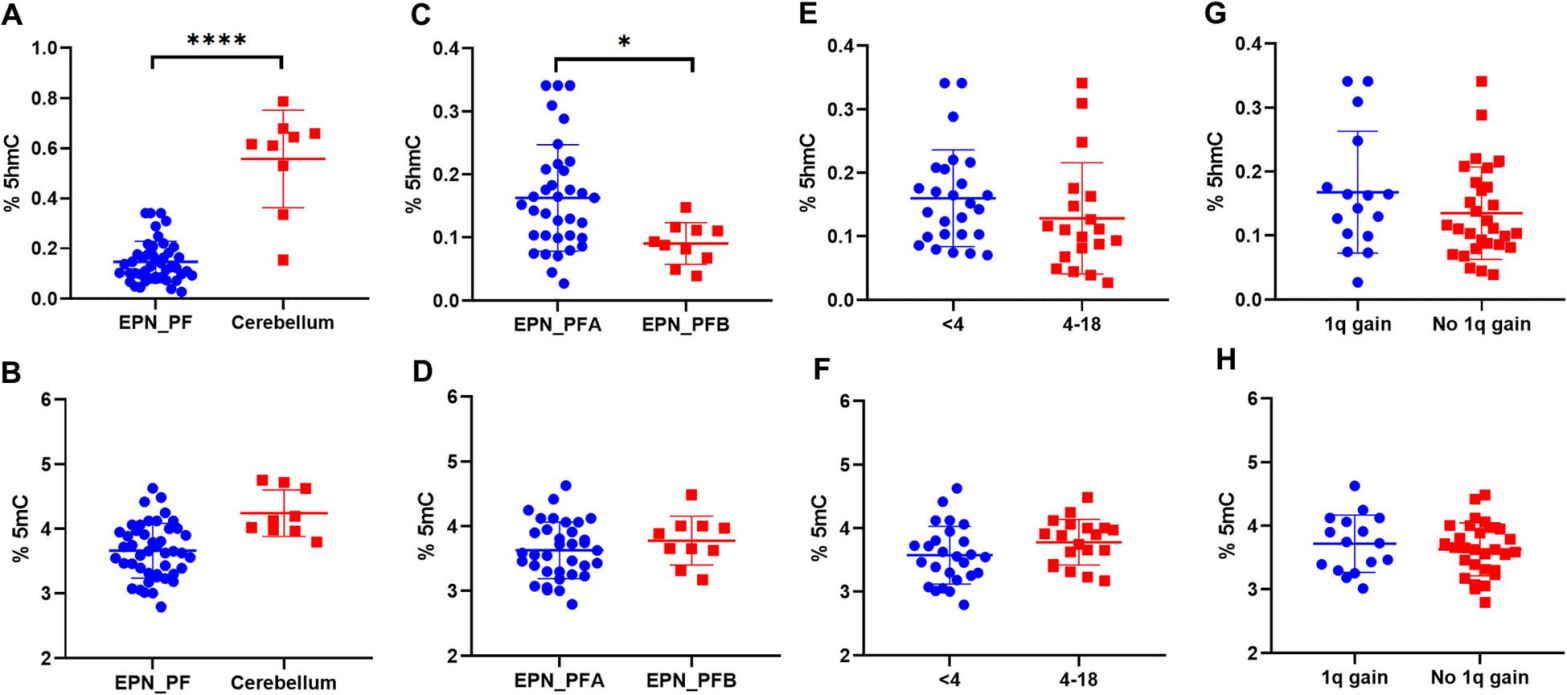
Comparative evaluation of 5hmC levels and 5mC levels as measured by UHPLC-MS/MS was respectively analyzed between tumors and normal cerebellum (**a**, **b**), two molecular subgroups (**c**, **d**), two age groups (**e**, **f**) and two subtypes of 1q status in (**g**, **h**). Bars, standard deviation. *P < 0.05; ****P < 0.0001. 5hmC by Mann–Whitney *U* test, 5mC by Student’s *t* test

To evaluate the global changes of 5hmC and 5mC levels in pediatric EPN_PF, we first performed the UHPLC-MS/MS analysis to measure global 5hmC and 5mC levels in 45 EPN_PF and 9 normal cerebellum samples. We found that 5hmC levels significantly decreased in tumor samples compared with those in normal cerebellum tissues [EPN_PF vs. cerebellum, 0.127 (0.028–0.341) vs. 0.617 (0.154–0.788); *P* < 0.001] (Fig. 1a). No significant difference of global 5mC levels was observed between EPN_PF and cerebellum (EPN_PF vs. cerebellum, 3.664 ± 0.426 vs. 4.245 ± 0.361; *P* = 0.653) (Fig. 1b).

### 5hmC levels were different between two molecular subgroups

We next compared the differences of 5hmC and 5mC levels between two molecular subgroups. As shown in Fig. 1c, EPN_PFA exhibited higher 5hmC levels than PFB [EPN_PFA vs. EPN_PFB, 0.152 (0.028–0.341) vs. 0.091 (0.039–0.148); *P* = 0.007]. No significant difference of 5mC levels between the two molecular subgroups (EPN_PFA vs. EPN_PFB, 3.630 ± 0.438 vs. 3.780 ± 0.379; *P* = 0.334) (Fig. 1d). Moreover, we found that the 5hmC levels and 5mC levels were similar between the age groups and subtypes of 1q status (Fig. 1e–h).

### 5hmC levels positively correlate with cell proliferation

**Fig. 2:**
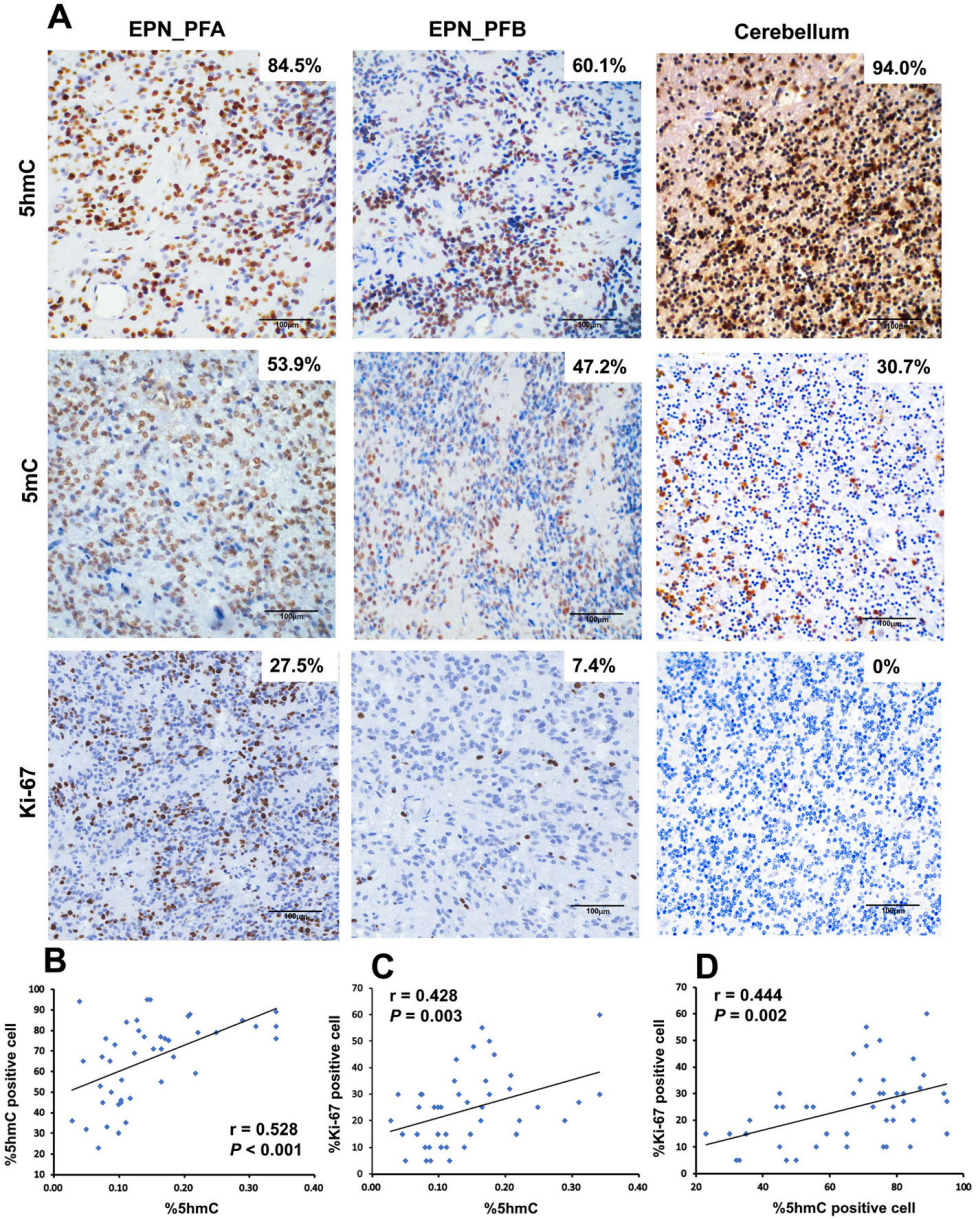
5hmC, 5mC, and Ki-67 index IHC staining in EPN_PF samples and normal cerebellum. **a** Representative image of 5hmC, 5mC, and Ki-67 staining in EPN_PF and cerebellum. **b** The Pearson correlation between nuclear positive cells of 5hmC and global 5hmC levels. **c** The Pearson correlation between Ki-67 index cells and global 5hmC levels. **d** The Pearson correlation between Ki-67 index and nuclear positive cells of 5hmC. Scale bars represent 100 μm

Furthermore, IHC staining yielded the similar previous results (Fig. 2a). The EPN_PF samples showed a lower nuclear positivity of 5hmC antibody (65.4% ± 19.1%) compared to the normal cerebellums (90% ± 4%, *P* = 0.004). The Pearson correlation analysis showed there was a significantly positive correlation between 5hmC positive cells percentage and the amount of 5hmC levels (*r* = 0.528, *P* < 0.001) (Fig. 2b). We next determine the relationship between 5hmC levels and cell proliferation using Ki-67 staining in EPN_PF. We found the tight correlation between 5hmC positive cells and Ki-67 index (*r* = 0.444, *P* = 0.002) (Fig. 2c). The results also showed that 5hmC levels were positively related with Ki-67 index (*r* = 0.428, *P* = 0.003) (Fig. 2d).

### High 5hmC levels related to poor prognosis

In this study, the median follow-up period was 38 months (range 6–60 months). At the endpoint of the follow-up analysis, 25 (55.6%) patients presented recurrence or progressive disease. Twenty-three (51.1%) patients were dead occurred during the data collection period. The estimated 3-year PFS and OS rates were 49.9 ± 8.1% (95% CI, 34.1–65.7%) and 48.8 ± 8.4% (95% CI, 32.3–65.3%), respectively.

**Fig. 3:**
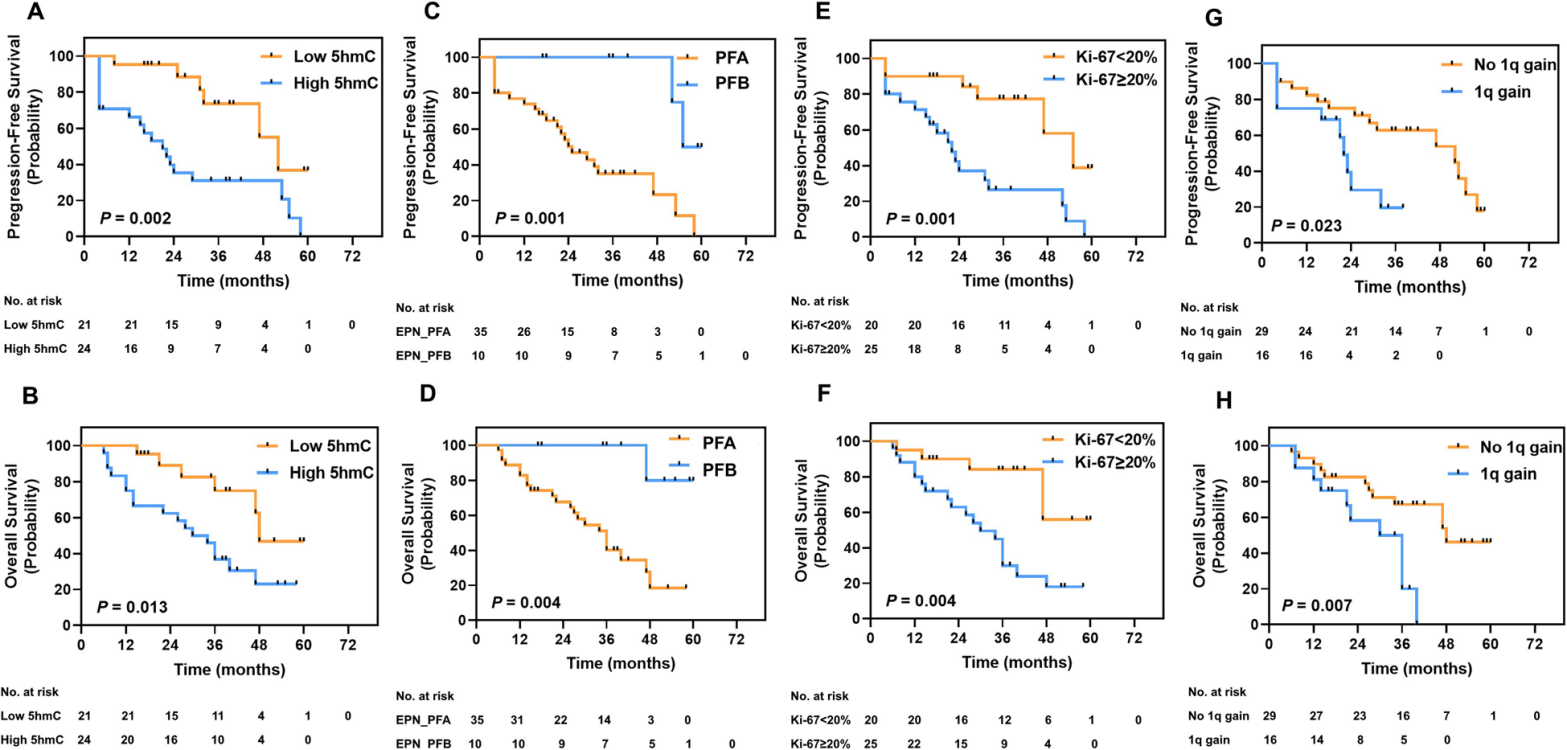
Kaplan–Meier survival analysis comparing PFS and OS for **a**, **b** low 5hmC group vs. high 5hmC group. **c**, **d** EPN_PFA vs. EPN_PFB. **e**, **f** Low Ki-67 index group vs. high Ki-67 index group. **g**, **h** No 1q gain group vs. 1q gain group. *P* value determined using the log-rank test

To further clarify the relationship between 5hmC level and prognostic factors, we divided patient cohorts into two subgroups according to 5hmC levels (UHPLC-MS/MS) [low 5hmC levels (< 0.102%) and high 5hmC levels (> 0.102%)] based on the Cut-off Finder [19]. We also dichotomized the Ki-67 index as high Ki-67 index (≥ 20%) and low Ki-67 (< 20%), as previous described [20]. We found that patients with low 5hmC levels (3-year PFS, 73.7 ± 11.5%; 3-year OS, 75.0 ± 11.1%) showed more favorable prognosis than those with high 5hmC levels (3-year PFS, 31.0 ± 9.7%, *P* = 0.002; 3-year OS, 36.7 ± 10.0%, *P* < 0.001; Fig. 3a, b). Patients with PFA had the worse survival rates (3-year PFS, 35.1 ± 9.0%; 3-year OS, 51.1 ± 8.9%), compared with patients with PFB (3-year PFS, 100%, *P* = 0.001; 3-year OS, 100%, *P* = 0.004; Fig. 3b, c). Patients with high Ki-67 index (3-year PFS, 26.5 ± 9.9%; 3-year OS, 45.0 ± 10.4%) had poorer prognosis compared to patients with low Ki-67 index (3-year PFS, 77.5 ± 10.0%, *P* = 0.001; 3-year OS, 84.0 ± 8.5%, *P* = 0.004; Fig. 3e, f). Moreover, 1q gain EPN_PF exhibited significantly worse PFS and OS as compared to no 1q gain EPN_PF (3-year PFS, 19.6% ± 12.2% vs. 62.8% ± 9.4%, *P* = 0.023; 3-year OS, 20.0 ± 12.3% vs. 67.3% ± 9.0%, *P* = 0.008; Fig. 3g, h).

### 5hmC level was an independent prognostic factor

On univariate analysis (Table 2), EPN_PFA (PFS: HR = 8.012; 95% CI, 1.815–35.371, *P* = 0.006, OS: HR = 10.854; 95% CI, 1.446–81.465; *P* = 0.020) and high 5hmC levels (PFS: HR = 3.438; 95% CI, 1.367–8.646; *P* = 0.009, OS: HR = 3.030; 95% CI, 1.192–7.702; *P* = 0.020) as well as 1q gain (PFS: HR = 2.666; 95% CI, 1.086–6.548; *P* = 0.032, OS: HR = 3.148; 95% CI, 1.283–7.722; *P* = 0.012) were associated with worse PFS and OS. Patients with high Ki-67 index were associated with worse PFS (HR = 3.726; 95% CI, 1.475–9.427; *P* = 0.005) but not for OS (*P* = 0.086).

**Table 2:** Univariate cox analysis for progression-free survival and overall survival for pediatric EPN_PF

	Progression-free survival			Overall survival		
Variable	Hazard ratio	95% CI	*P* value	Hazard ratio	95% CI	*P* value
Mo. subgroup (EPN_PFA vs. EPN_PFB)	8.012	1.815–35.371	*0.006*	10.854	1.446–81.465	*0.020*
5hmC subgroup (high vs. low)	3.438	1.367–8.646	*0.009*	3.030	1.192–7.702	*0.020*
Resection (STR vs. GTR)	1.338	0.602–2.972	0.475	1.445	0.634–3.292	0.381
Ki-67 index (≥ 20% vs. < 20%)	3.726	1.475–9.427	*0.005*	1.787	0.921–3.468	0.086
1q gain (yes vs. no)	2.666	1.086–6.548	*0.032*	3.148	1.283–7.722	*0.012*
Age, years (< 4 vs. 4–18)	1.567	0.678–3.618	0.293	1.376	0.580–3.262	0.469
Radiotherapy (yes vs. no)	0.695	0.281–1.717	0.430	0.477	0.197–1.159	0.102
Chemotherapy (yes vs. no)	0.839	0.334–2.112	0.710	0.226	0.052–0.985	0.053
Histology (WHO III vs. WHO II)	2.115	0.760–5.885	0.151	2.938	0.855–10.104	0.087
Gender (male vs. female)	1.416	0.530–3.783	0.487	1.015	0.400–2.577	0.975

*P* values in italics are of statistical significance

*Abbreviations*: *Mo.* molecular, *PF* posterior fossa, *STR* subtotal resection, *GTR* gross total resection, *EPN_PFA* Group A posterior fossa ependymoma, *EPN_PFB* Group B posterior fossa ependymoma

A multivariate Cox proportional hazards model with backward stepwise selection for PFS and OS was fitted using the following potential prognostic factors (based on univariate analysis and published literature [21, 22]): molecular subgroup, 5hmC subgroup, Ki-67 index, 1q gain, and extent of resection. As seen in Table 3, EPN_PFA molecular subgroup (PFS: HR = 5.253; 95% CI, 1.052–26.237; *P* = 0.043, OS: HR = 7.496; 95% CI, 0.928–60.557; *P* = 0.048) and high 5hmC levels (PFS: HR = 3.014; 95% CI, 1.040–8.738; *P* = 0.042, OS: HR = 2.788; 95% CI, 0.974-7.982; *P* = 0.047) were confirmed as independent inferior prognostic indicators for PFS and OS. STR were confirmed as independent inferior prognostic indicators for OS (HR = 2.682; 95% CI, 1.168–7.664; *P* = 0.039). Age, WHO grade, Ki-67 index, and adjuvant treatment exhibited no association with PFS and OS.

**Table 3:** Multivariate cox analysis for progression-free survival and overall survival for pediatric EPN_PF

	Progression-free survival			Overall survival		
Variable	Hazard ratio	95% CI	*P* value	Hazard ratio	95% CI	*P* value
Mo. subgroup (EPN_PFA vs. EPN_PFB)	5.253	1.052–26.237	*0.043*	7.496	0.928–60.557	*0.048*
5hmC subgroup (high vs. low)	3.014	1.040–8.738	*0.042*	2.788	0.974–7.982	*0.047*
Resection (STR vs. GTR)	2.292	0.919–5.719	0.075	2.682	1.168–7.664	*0.039*

*P* values in italics are of statistical significance

*Abbreviations*: *Mo.* molecular, *STR* subtotal resection, *GTR* gross total resection, *EPN_PFA*Group A posterior fossa ependymoma, *EPN_PFB* Group B posterior fossa ependymoma

Furthermore, we attempted to validate whether 5hmC level was an independent prognostic factor within EPN_PFA children alone (*n* = 35). A multivariate analysis revealed that 5hmC levels and extent of resection were independent predictors of OS in children with EPN_PFA, but none of variables showed significant association with PFS (Additional file 1: Table S1).

## Discussion

Epigenetic modifications are important in normal development and frequently alter during tumorigenesis [23]. Recent studies have demonstrated that the loss of 5hmC in different types of cancers might play an essential role in pathogenesis [24–28]. Kraus et al. [29] reported that loss of 5hmC was found in EPN, but only one case (1/23) in this group was EPN_PF. However, it is still unclear whether level of 5hmC is changed in pediatric EPN_PF. To the best of our knowledge, this is the first study to illustrate the alternation of 5hmC level in EPN_PF. We showed that global levels of 5hmC are strikingly decreased in pediatric EPN_PF tumor tissues compared with normal cerebellum tissues. Our results further support the previous observations of the reduced level of 5hmC that occurred in other types of CNS tumors [14, 17, 29]. Moreover, we found that 5hmC score based on IHC staining with 5hmC antibody significantly related to 5hmC levels detected by UHPLC-MS/MS, suggesting that the IHC staining approach may be a useful method to identify 5hmC level in pediatric EPN_PF.

The limited number of biomarkers reliably predicting prognosis in EPN highlights the importance of developing more robust prognostic markers [22]. Previous studies indicate that 5hmC levels are associated with clinical outcomes in multiple types of cancers [27, 30]. Analysis of multiple cohorts of intracranial ependymoma highlights a wide variance in the utility of the grade II versus grade III distinction as a prognostic marker. However, the utility of histological grading of ependymoma for risk stratification has been controversial and without consistent associations of tumor grade with patient outcome [11]. Recently, DNA methylation patterns and DNA copy number profiles can be used to predict clinical outcomes for patients with EPN [22]. In this study, our result indicated that high 5hmC level is an independent prognostic factor of poor survival in pediatric EPN_PF on PFS and OS. In most solid tumors, low 5hmC levels relate to high tumor grade and worse outcomes [17, 27, 29, 31–33]. However, our results are in line with the previous report that high levels of 5hmC independently correlate with inferior overall survival in acute myeloid leukemia [34] and suggest that 5hmC may be involved in the distinct mechanisms of tumorigenesis and malignant transformation. These findings remain to be confirmed in future studies.

Recent advances in the biological characterization of EPN_PF have demonstrated the existence of two clinically, demographically, and molecularly distinct entities [4]. EPN_PFA tumors show higher methylation of CpGi. EPN_PFA patients are younger, have laterally located tumors with an increased occurrence of chromosome 1q gain, and behave more aggressively compared with EPN_PFB patients [4]. In addition to CpGi hypermethylation, DNA hypomethylation and global H3K27me3 reduction in the absence of recurrent genetic changes in EPN_PF suggests that epigenetic mechanisms are central to EPN_PF pathogenesis [7, 8]. Moreover, several studies [7, 8, 35–37] showed that reduced H3K27me3 in EPN_PF is not genetically driven but epigenetically deregulated. Our study firstly observed distinct 5hmC levels between two molecular subgroups, suggesting that 5hmC may participate in the abnormal DNA methylation in pediatrics EPN_PF. Future researchers on EPN_PF should focus on the mechanism of epigenetic alternations.

We found a strong positive correlation between 5hmC levels and Ki-67 index in pediatric EPN_PF. Several studies demonstrate that higher Ki-67 index seems to be associated with poor prognosis in pediatrics EPN [20, 38, 39]. Our data confirm previous results and further suggest that high 5hmC levels associated with inferior outcomes. However, some studies have reported that 5hmC levels are inverse correlation of cellular proliferation in different types of cancers [17, 40–42]. These discrepancies may be explained by thedistinct tumorigenesis in this benign tumor compared with other malignant tumors. However, the mechanism of 5hmC influenced tumor cell proliferation in EPN_PF needs further research.

Our study had several limitations. The first limitation of our study was the small sample size and the relatively short follow-up period limited our ability to detect robust survival predictors. Future studies with large sample sizes and long-term follow-up are needed to confirm the results of our findings. Second, the molecular subgroup was classified by IHC. To overcome this limitation, future studies using fresh frozen tumor tissues followed by methylation arrays are needed [4].

## Conclusions

In our study, we found that 5hmC is a potential prognostic predictor that may contribute to the improvement of clinical risk stratification for EPN_PF. Our results indicate that the characteristic of 5hmC level is associated with molecular subgrouping and cell proliferation. These findings suggest that the mechanisms responsible for regulating 5hmC may represent a possible future therapeutic target.

## Methods

### Study design and samples

The main objective of this study was to assess the clinical characteristics of DNA hydroxymethylcytosine in pediatric EPN_PF. We used UHPLC-MS/MS to investigate 5hmC abundance in EPN_PF. Further, we conducted molecular classification using IHC. A total of 45 patients (age < 18 years) who were diagnosed with EPN_PF at Beijing Tiantan Hospital between January 2010 and December 2017 were included in this study. Clinical data, including age at diagnosis, gender, tumor size, treatment, recurrence data, and survival, were collected by retrospective chart review. Two neuropathologists reviewed the histopathologic findings according to 2016 WHO classification of CNS tumors [1]. Follow-up evaluations were performed on all patients via either an outpatient consultation or a telephone interview. This study was approved by the ethics committee of Beijing Tiantan Hospital, Capital Medical University. Written informed consent was obtained.

Tumor tissues were obtained during initial surgery before radiation or any other adjuvant treatment. All samples were snap-frozen (− 80 °C) or fixed with 4% buffered formalin, paraffin-embedded. All tumor specimens were sterilely stored at Beijing Neurosurgical Institute by the Ethics Review Board of the Beijing Tiantan Hospital. As control samples, all normal cerebellums used in this study were provided by the Human Brain Bank, Chinese Academy of Medical Sciences & Peking Union Medical College, with the approval from the Institutional Review Board of the Institute of Basic Medical Sciences, Chinese Academy of Medical Sciences (Approval Number: 009-2014).

### Definition of EPN_PF molecular subgroup by immunohistochemistry

IHC analyses were performed as reported [9]. In brief, tissue sections were cut at 5 μm, followed by deparaffinization and rehydration using xylene and ethanol. Then, the slides were incubated in 3% hydrogen peroxide for 10 min in phosphate-buffered saline to block endogenous peroxidase activity. Slides were incubated overnight with rabbit monoclonal anti-H3K27me3 antibody (C36B11, Cell Signaling, Danvers, MA, USA) at a concentration of 1:150 using the standard Leica Bond protocol IHC-F. The Leica Bond Polymer Refine DAB detection kit was used according to the manufacturer’s instructions. All IHC slides were evaluated by two independent neuropathologists; the scoring methods were performed as report described [9]. H3K27me3 positive staining was defined as scored positive when more than 80% cells had nuclear positivity and scored negative when they did not.

### Evaluation of global 5mC and 5hmC by UHPLC-MS/MS

The absolute amount of 5hmC and 5mC in EPN was measured as previously described [43, 44]. Briefly, DNA isolation was performed using the Wizard® Genomic DNA Purification Kit (A1620, Promega, Madison, WI, USA) according to the manufacturer’s protocol. DNA for each sample (1 μg/sample) was denatured by heating at 100 °C for 3 min and then digested by incubation at 42 °C with nuclease P1 (2U, Sigma, N8630, Darmstadt, Germany) for 6 h. Subsequently, 1 U of alkaline phosphatase (Sigma, M183A) was added and incubated at 37 °C for another 6 h. Finally, the sample was diluted to a total volume of 60 μl and filtered (0.45 μm, PALL). Nucleosides were separated by ultra-performance liquid chromatographic on T3 column (WATERS, 186003538, MA, USA) and detected using triple-4 quadrupole mass spectrometer (WATERS, ACQUITY UPLC XEVO TQ-S). The mass transitions of m/z 228.4 to 112.2 (C), m/z 242.3 to 126.1 (mC), m/z 258.2 to 124.2 (hmC) were monitored and recorded. Quantification was performed in comparison with the standard curve obtained from pure nucleoside standards running on the same batch of samples. Finally, the percentages of 5mC and 5hmC were calculated by the following formula: M (cytosine) and M (5mC) are the molar quantities of cytosine: 5mC% = M (5mC)/[M (cytosine) + M (5mC)] × 100, 5hmC% = M (5hmC)/[M(cytosine) + M(5mC)] × 100.

### IHC analysis for 5hmC, 5mC, and Ki-67

The utilized primary antibodies were including 5hmC (1:800, ab214728, Abcam), 5mC (1:200, ab10805, Abcam), and Ki-67 (1:1500, ab15580, Abcam). Immunohistochemical detection of 5hmC and 5mC was performed as described above exclusive of the step of DNA denatures by 2N HCl [27]. The 5hmC and Ki-67 staining and score methods were performed according to described previously [45]. In brief, positive staining was defined as a dark brown staining pattern, confined to the nuclear region. Scant or fine granular background staining or no staining was considered as negative. The mean value of the five snapshots was calculated to represent the percentage of positive cells in each case.

### 1q gain by interphase FISH

Dual color interphase fluorescence in FISH analysis was performed on formalin-fixed paraffin-embedded sections as previously described using commercially available 1q25 (spectrum green) and 1p36 (spectrum orange) probe sets (ZytoVision, Germany) [46]. The evaluation criteria and scoring system adopted was based on previously described [46, 47].

### Statistical analysis

All statistical analyses were performed with SPSS 23 (IBM Corp., New York, NY, USA) and two-sided *P* values < 0.05 were considered statistically significant. The normality of variables was assessed. Data are expressed as mean ± standard deviation (SD) or median (minimum to maximum). Differences in mean and median values were evaluated by using Student’s *t* test and the Mann–Whitney *U* test, respectively. Associations between categorical variables were assessed via Fisher’s exact test. To interpret the effect of 5hmC level in a more clinically relevant manner, 5hmC levels were dichotomized into two groups using Cutoff Finder [19]. The cutoff values (0.102%) were defined as the points with the most significant split between groups, including PFS and OS.

For the survival analysis, overall survival (OS) was calculated from the date of initial surgery that established the pathological diagnosis to the time of death. Progression-free survival (PFS) was derived from the date of initial surgery to the time of progression. Kaplan–Meier curves of OS and PFS were generated and log-rank tests were used to compare OS and PFS between demographic and clinical factors. Multivariate Cox proportional hazards regression models with backward stepwise selection were used to identify significant prognostic factors for OS and PFS. Hazard ratios with corresponding 95% confidence intervals were calculated.

## Supplementary information

**Additional file 1: Figure S1**, **S2**, **Table S1**. Supplementary Material

## Data Availability

The datasets used and/or analyzed during the current study are available from the corresponding author on reasonable request.

## Abbreviations

5hmC: 5-Hydroxymethylcytosine
5mC: 5-Methylcytosine
CNS: Central nervous system
CpGi: CpG island
EPN: Ependymoma
EPN_PF: Posterior fossa ependymoma
EPN_PFA: Group A posterior fossa ependymoma
EPN_PFB: Group B posterior fossa ependymoma
FISH: Fluorescence in situ hybridization
GTR: Gross total resection
IHC: Immunohistochemistry
OS: Overall survival
PFS: Progression-free survival
STR: Subtotal resection
UHPLC-MS/MS: Ultra-high-performance liquid chromatography-mass spectrometry
