# Supplementary material for: Characterization of global 5-hydroxymethylcytosine in pediatric posterior fossa ependymoma
Source: Clin Epigenetics. 2020 Jan 28;12:19. doi: 10.1186/s13148-020-0809-8 (PMC6988368; doi:10.1186/s13148-020-0809-8)
Supplement: Supplementary file 1 — Additional file 1: Figure S1, S2, Table S1. Supplementary Material [file 13148_2020_809_MOESM1_ESM.docx]

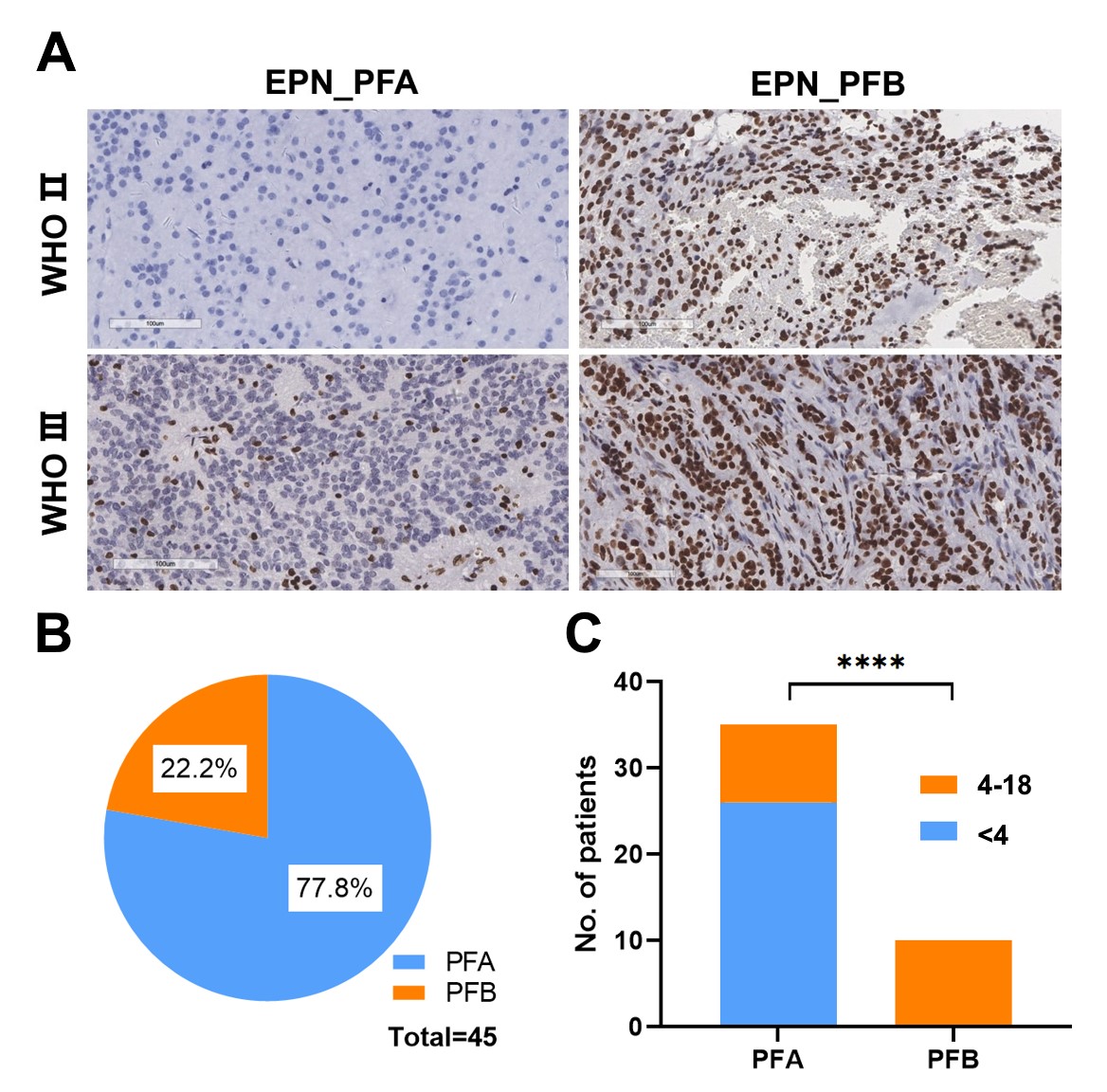


**Figure S1**. H3K27me3 immunohistochemistry segregates group A posterior fossa ependymoma (EPN_PFA) from group B posterior fossa ependymoma (EPN_PFB). **A.** Representative image of H3K27me3 immunostaining in EPN_PFA and EPN_PFB. **B.** Proportions of patients with two molecular subgroups in our cohort. **C.** Different proportion of patients with age < 4 years or 4-18 years between two molecular subgroups. ****, *P* < 0.0001. By Fisher’s exact test.


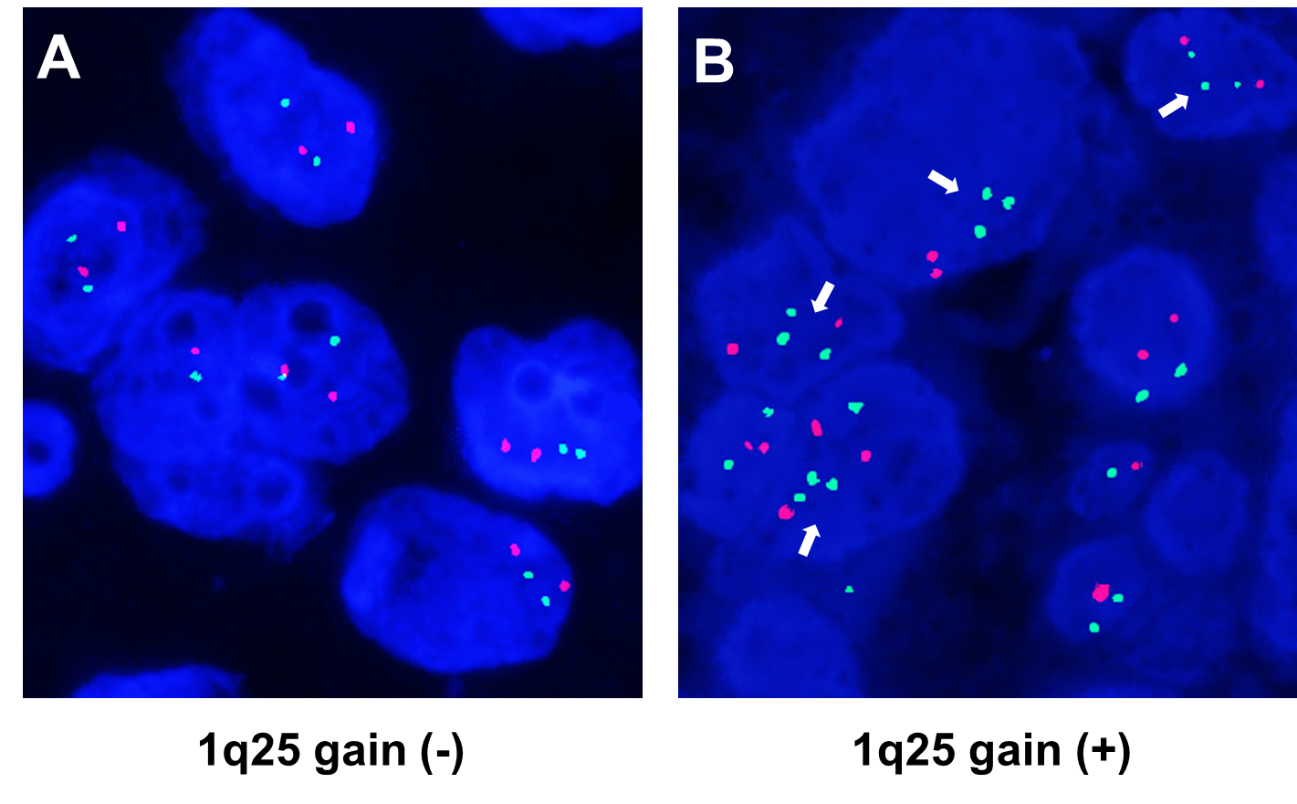


**Figure S2.** 1q status analysis by fluorescence in situ hybridization (FISH; test signal: 1q25 green fluorochrome, control signal: 1p36 red fluorochrome). **A**. Representative image of 1q gain (-) showing two green signals with two red signals. **B**. Representative image of 1q gain (+) showing three green signals (white arrows) and two red signals.

**Table S1.** Multivariate cox analysis for progression-free survival and overall survival for pediatric EPN_PFA (n = 35)

| Variable | Progression-Free Survival | | | Overall Survival | | |
| --- | --- | --- | --- | --- | --- | --- |
|  | HR | 95% CI | *P* | HR | 95% CI | *P* |
| 5hmC subgroup (High vs. Low) | 2.670 | 0.912-7.823 | 0.073 | 2.969 | 0.976-9.026 | *0.048* |
| Resection (STR vs. GTR) | 2.095 | 0.817-5.375 | 0.124 | 3.117 | 1.190-8.161 | *0.021* |
| 1q gain (Yes vs. No) | 1.456 | 0.593-3.576 | 0.413 | 1.810 | 0.732-4.478 | 0.199 |

*P* values in italics are of statistical significance

*Abbreviation*: *STR*, subtotal resection; *GTR*, gross total resection
